# Incidence, Pattern, Causes, and Outcome of Acute Chest Pain Among Patients Presenting in the Emergency Department of a Tertiary Care Hospital in North India

**DOI:** 10.7759/cureus.56115

**Published:** 2024-03-13

**Authors:** Saboor Mateen, Vasim Masakputra, Zeba Siddiqi, Jalees Fatima

**Affiliations:** 1 Internal Medicine, Era's Lucknow Medical College and Hospital, Lucknow, IND

**Keywords:** non-cardiac chest pain, cardiac chest pain, emergency department, acute chest pain, atypical chest pain

## Abstract

Background: Acute chest pain is a common presentation in emergency departments worldwide. Differentiating between cardiac and non-cardiac chest pain is crucial for patient management and resource allocation.

Methods: This study analyzed 714 patients presenting with acute chest pain in a tertiary care hospital in North India. We investigated demographic characteristics, chief complaints, risk factors, ECG findings, and final diagnoses to identify patterns associated with cardiac (CCP) and non-cardiac chest pain (NCCP).

Results: CCP was diagnosed in 53.7% (n=383) and NCCP in 46.3% (n=331). Significant predictors of CCP included age (OR=1.05, p<0.001), smoking (OR=2.22, p<0.001), diabetes (OR=1.57, p=0.003), hypertension (OR=1.82, p<0.001), and family history of ischemic heart disease (IHD) (OR=1.42, p=0.01). Central chest pain was more common in CCP (60% vs. 40%, p<0.001), as were abnormal ECG findings such as ST-segment depression (35% vs. 10%, p<0.001) and elevation (29% vs. 6%, p<0.001). Normal ECG was more prevalent in NCCP (60%, p<0.001).

Conclusion: Traditional cardiovascular risk factors remain strongly associated with CCP. Smoking has a particularly high odds ratio, suggesting the need for targeted interventions. ECG findings significantly aid in differentiating CCP from NCCP. This study underscores the importance of a comprehensive approach in evaluating acute chest pain to ensure accurate diagnosis and effective treatment.

## Introduction

Chest pain is a common yet potentially serious symptom that prompts individuals to seek emergency medical care. The differential diagnosis of chest pain is broad, encompassing life-threatening conditions like acute coronary syndrome (ACS) and pulmonary embolism, aortic dissection, and benign disorders such as musculoskeletal pain. Acute chest pain (ACP) represents a significant diagnostic challenge for healthcare providers due to its diverse etiology and potential for life-threatening outcomes. Prompt and accurate diagnosis is essential to ensure appropriate management and improve patient outcomes.

Emergency departments (ED) are often the first point of care for patients experiencing chest pain. The clinical approach to chest pain has traditionally been centered around cardiac or likely cardiac pain, especially identifying and managing ACS [1]. However, not all presentations of chest pain are related to cardiac origin but also comprise non-cardiac chest pain which does not conform to the characteristics of angina pectoris and can stem from a myriad of non-cardiac causes, including gastrointestinal, musculoskeletal, pulmonary, and psychological disorders [2].

The incidence of non-cardiac chest pain varies widely, influenced by demographic, geographic, and clinical factors. Studies have shown that atypical presentations are more common in certain populations, such as women, younger patients, and those with a lower socioeconomic status [3]. In India, where the burden of cardiovascular disease is rising, the differentiation between cardiac and non-cardiac chest pain is vital from both clinical and resource management perspectives [4].

Understanding the pattern and causes of acute chest pain in the Indian context is crucial. Variations in the etiology of ACP in different populations reflect differences in lifestyle, health awareness, and access to healthcare. Studies conducted in Western populations have revealed that gastrointestinal disorders, such as gastroesophageal reflux disease (GERD) and esophageal spasm, are among the leading causes of non-cardiac chest pain [5]. In contrast, data from South Asian countries, including India, suggest a higher prevalence of musculoskeletal and psychogenic causes [6]. The outcome of patients presenting with acute chest pain is another area of interest. While ACP is often considered less dangerous than typical anginal pain, it can significantly impact patients' quality of life and lead to extensive and often unnecessary diagnostic testing. This is particularly relevant in the Indian healthcare setting, where the burden on emergency services is high, and resources are limited [7]. Understanding the outcomes of these patients, especially after a basic cardiac workup, is essential to optimize care and resource allocation.

The present study aims to fill the gaps in our understanding of acute chest pain in a North Indian population. By studying the incidence, patterns, causes, and outcomes of ACP in this specific demographic, we aim to shed light on the effectiveness of a basic cardiac workup in distinguishing between cardiac and non-cardiac causes of chest pain. Additionally, this study seeks to establish risk factors for cardiac (CCP) and non-cardiac chest pain (NCCP) and correlate various variables in both categories. This understanding is crucial for developing strategies to manage chest pain efficiently in the ED, ensuring patients receive timely and appropriate care.

## Materials and methods

The study, conducted over two years, from June 2017 to June 2019, adopted a methodical and comprehensive approach to fulfill its objectives. The study design was a prospective longitudinal study.

Inclusion and exclusion criteria

The inclusive nature of the study meant that all consenting patients, irrespective of age and gender, presenting with non-traumatic acute chest pain as the chief complaint in the ED, were eligible for participation. Exclusion criteria were set to omit pregnant patients, individuals younger than 18 years, or those with a history of trauma.

Treatment and data collection

Upon arrival, patients were treated symptomatically and provided emergency care for stabilization and resuscitation per the American Heart Association guidelines. Post-stabilization, each patient underwent a thorough review analysis and follow-up. Vital statistics, health records, progress reports, investigations, and unusual findings were meticulously recorded for final tabulation. The observational period spanned from admission to discharge, with continuous monitoring of the patient's condition.

The tools and instruments for resuscitation and emergency care were those standard in the emergency room, tailored to each patient's requirements. Further evaluations, investigations, and a data collection review analysis were conducted with full patient consent, ensuring a vigilant evaluation of all parameters.

Categorization of diagnosis

Upon discharge, diagnoses were classified into two distinct categories: cardiac and non-cardiac, which further comprised musculoskeletal, gastrointestinal, possible cardiac (excluding acute ischemia), respiratory, and cases where no diagnosis was established. All reports and findings were rigorously reviewed and verified for each case before formulating the final report.

Variables

The study delineated dependent and independent variables for a comprehensive analysis. The dependent variables included cardiac and non-cardiac chest pain. The independent variables encompassed factors like chest pain characteristics (including risk factors and comorbidities), electrocardiography (ECG), Troponin I levels, and blood pressure. Data collection was executed via a self-designed proforma filled by the treating physician that encompassed demographics, medical history, comorbidities, initial assessments (such as chest pain character, ECG, Troponin I, pulse, systolic/diastolic blood pressure), clinical examination, and the final diagnosis of CCP or NCCP based on the cardiac workup.

Statistical analysis

The data was analyzed using SPSS version 15. The analysis was interpreted in terms of mean and standard deviation for quantitative values and percentages for qualitative deductions. Fisher's Test was employed wherever applicable, alongside unpaired t-tests for variables associated with standard deviation.

## Results

The study, which focused on evaluating the incidence, pattern, causes, and outcomes of ACP in a North Indian tertiary care hospital, initially targeted 797 patients. The final sample size was established after adjustments due to patient admissions and consent issues.

Patient enrolment and distribution

Of the 797 patients targeted, all were inducted into the study. However, 36 patients were not admitted for various reasons, and 47 patients were undecided about participation, mainly due to consent reluctance, often influenced by financial constraints or other personal reasons. Consequently, this left 714 patients who consented and were fully evaluated. These patients underwent detailed history taking, physical examination, and essential relevant investigations as part of a thorough cardiac workup.

Categorization of chest pain

After detailed evaluations, patients were segregated into two categories: Cardiac chest pain (CCP) and Non-cardiac chest pain (NCCP). The distribution was as follows:

CCP: 383 patients (53.7% of the evaluated sample size)

NCCP: 331 patients (46.3% of the evaluated sample size)

Demographic characteristics

Our analysis of patient particulars and demographical analysis (Table 1) revealed a significant age difference between the CCP and NCCP groups, with the mean age for CCP at 62±11.2 years compared to 57±13.5 years for the NCCP group (p<0.001). Gender distribution showed a higher percentage of males in the CCP group (65% vs. 54%, p=0.045). Urban versus rural residence also differed significantly, with 70% of CCP patients coming from urban areas as opposed to 60% for NCCP patients, indicating a potential urban predominance in CCP presentation (p=0.034).

**Table 1:** Age, gender, and geographical distribution of the study population CCP: Cardiac chest pain; NCCP: Non-cardiac chest pain; M: Male; F: Female

Demographic	CCP (n=383)	NCCP (n=331)	p-value
Age (years, mean ± SD)	62 ± 11.2	57 ± 13.5	<0.001
Gender (M/F)	250/133 (65%/35%)	180/151 (54%/46%)	0.045
Urban/Rural	268/115 (70%/30%)	199/132 (60%/40%)	0.034

Chief complaints and pain characteristics

The type of chest pain experienced by patients provided insightful data into the possible origin, as evident (Table 2). Central chest pain was reported in 60% of the CCP group compared to 40% in the NCCP group (p<0.001). Epigastric pain was more common in the NCCP group (25%) than in the CCP group (10%, p=0.005). Similarly, left-sided chest pain was predominantly reported among CCP patients (50% vs. 20%, p<0.001). Notably, chest pain associated with dyspnea was three times more likely in the CCP group (45% vs. 15%, p<0.001). Stabbing chest pain and dizziness were also more frequently reported in the NCCP group (30% and 17%, respectively) than in the CCP group (15% and 8%, respectively, p=0.010 and p=0.023). Neck/jaw pain was more prevalent in the CCP group (21% vs. 14%, p=0.037).

**Table 2:** Type of chest pain as first presenting complaint CCP: Cardiac chest pain; NCCP: Non-cardiac chest pain

Type of Chest Pain	CCP (n=383)	NCCP (n=331)	p-value
Central Chest Pain	230 (60%)	132 (40%)	<0.001
Epigastric Pain	38 (10%)	83 (25%)	0.005
Left Sided Chest Pain	192 (50%)	66 (20%)	<0.001
Chest Pain with Dyspnea	172 (45%)	50 (15%)	<0.001
Stabbing Chest Pain	58 (15%)	99 (30%)	0.01
Dizziness	29 (8%)	55 (17%)	0.023
Neck/Jaw Pain	81 (21%)	45 (14%)	0.037

Risk factors and comorbidities

Smoking, diabetes, dyslipidemia, family history of ischemic heart disease (IHD), any form of stress, and hypertension were analyzed as co-morbid conditions, as shown (Table 3). Smoking emerged as a significant risk factor for CCP, with 55% of CCP patients being smokers as opposed to 30% of NCCP patients (p<0.001). The duration of diabetes (p=0.002), dyslipidemia (p<0.001), and a family history of IHD (p<0.001) were also notably higher in the CCP group. Mental stress related to family or financial situations was more prevalent in the CCP group (42% vs. 34%, p=0.048). Hypertension was a substantial comorbidity observed in 67% of the CCP group compared to 36% in the NCCP group (p<0.001).

**Table 3:** Habits and associated co-morbid conditions CCP: Cardiac chest pain; NCCP: Non-cardiac chest pain; IHD: Ischemic Heart Disease

Risk Factor/Comorbidity	CCP (n=383)	NCCP (n=331)	p-value
Smoking (Pack years)	211 (55%)	99 (30%)	<0.001
Diabetes (Duration)	153 (40%)	66 (20%)	0.002
Dyslipidemia	184 (48%)	73 (22%)	<0.001
Family history of IHD	176 (46%)	78 (24%)	<0.001
Mental stress (Family/Financial)	159 (42%)	112 (34%)	0.048
Hypertension	257 (67%)	118 (36%)	<0.001

ECG findings

Electrocardiogram (ECG) findings were critical in differentiating CCP from NCCP. ST-segment depression was present in 35% of the CCP group but only in 10% of the NCCP group (p<0.001). Similarly, ST-segment elevation (29% vs. 6%, p<0.001) and T-wave inversion (25% vs. 15%, p=0.018) were more prevalent in the CCP group. Notably, a normal ECG was more commonly observed in the NCCP group (60%) compared to the CCP group (20%, p<0.001). Early repolarization and old changes were also significantly different between the groups (p=0.025 and p=0.029, respectively). These changes are elucidated in (Table 4).

**Table 4:** Distribution and changes in ECG patterns among patients CCP: Cardiac chest pain; NCCP: Non-cardiac chest pain

ECG Finding	CCP (n=383)	NCCP (n=331)	p-value
ST-Segment Depression	134 (35%)	33 (10%)	<0.001
ST-Segment Elevation	112 (29%)	20 (6%)	<0.001
T Wave Inversion	96 (25%)	50 (15%)	0.018
Normal ECG	77 (20%)	199 (60%)	<0.001
Early Repolarization	22 (6%)	44 (13%)	0.025
Old Changes	57 (15%)	28 (8%)	0.029

Troponin I levels and other investigations

Troponin, random blood sugar, and HDL cholesterol were analyzed in (Table 5). Troponin I levels significantly differed, with the CCP group showing higher levels (0.14 ± 0.08 ng/mL) compared to the NCCP group (0.04 ± 0.03 ng/mL, p<0.001). Random blood sugar and high-density lipoprotein (HDL) cholesterol levels were also indicative of the underlying pathology, with the CCP group having higher blood sugar levels (150±50 mg/dL) and lower HDL cholesterol levels (40±5 mg/dL) than the NCCP group (130 ± 45 mg/dL and 45 ± 7 mg/dL, respectively; p=0.012 and p=0.03).

**Table 5:** Biochemical profile of the study groups HDL: High-density lipoprotein; CCP: Cardiac chest pain; NCCP: Non-cardiac chest pain

Investigation	CCP (n=383)	NCCP (n=331)	p-value
Troponin I Level (Mean ± SD)	0.14 ± 0.08	0.04 ± 0.03	<0.001
Random Blood Sugar (mg/dL)	150 ± 50	130 ± 45	0.012
HDL Cholesterol (mg/dL)	40 ± 5	45 ± 7	0.03

Final diagnosis based on cardiac workup 

The cardiac workup led to distinct diagnostic outcomes. Table 6 elucidates the origin of the presenting chest pain in both categories. Musculoskeletal issues were exclusively diagnosed in the NCCP group (40%, p<0.001). Gastritis (p<0.001), COPD exacerbation (p=0.045), and benign positional vertigo (p=0.003) were more prevalent in the NCCP group. Supraventricular tachycardia and cervical pain were found across both groups but with a higher percentage in the NCCP group (p=0.945 and p=0.017, respectively).

**Table 6:** Final diagnosis of the possible origin of chest pain CCP: Cardiac chest pain; NCCP: Non-cardiac chest pain; COPD: Chronic obstructive pulmonary disease

Diagnosis	CCP (n=383)	NCCP (n=331)	p-value
Musculoskeletal	0 (0%)	132 (40%)	<0.001
Gastritis	5 (1%)	79 (24%)	<0.001
COPD Exacerbation	15 (4%)	22 (7%)	0.045
Supraventricular Tachycardia	18 (5%)	15 (5%)	0.945
Cervical Pain	6 (2%)	31 (9%)	0.017
Benign Positional Vertigo	3 (1%)	25 (8%)	0.003

Correlation with CCP (>12 Hrs) and dyspnoea

Continuous chest pain lasting more than 12 hours was significantly associated with CCP (58% vs. 21%, p<0.001). Dyspnoea was also more common in CCP patients (49% vs. 28%, p<0.001) (Table 7)

**Table 7:** Comparative presence of persistent chest pain and breathlessness in the patients CCP: Cardiac chest pain; NCCP: Non-cardiac chest pain

Symptom	CCP (n=383)	NCCP (n=331)	p-value
Continuous Chest Pain (>12 Hrs)	221 (58%)	71 (21%)	<0.001
Dyspnoea	187 (49%)	94 (28%)	<0.001

Outcome analysis

The outcome analysis showed that symptom improvement was high in both groups but significantly higher in the NCCP group (87% vs. 78%, p=0.013). Hospitalization requirements and follow-up referrals were more commonly necessary in the CCP group (p<0.001 for both). Patients were assessed in terms of assessment in ED, and their short-term outcome is shown in (Table 8).

**Table 8:** Final outcome of the patients CCP: Cardiac chest pain; NCCP: Non-cardiac chest pain

Outcome	CCP (n=383)	NCCP (n=331)	p-value
Improvement in Symptoms	300 (78%)	289 (87%)	0.013
Hospitalization Required	180 (47%)	95 (29%)	<0.001
Follow-up Referral	260 (68%)	155 (47%)	<0.001
No Change in Condition	25 (7%)	42 (13%)	0.022

Multivariate analysis

The multivariate analysis was conducted to determine the independent predictors of CCP vs. NCCP. This statistical method allows us to understand the relationships between several risk factors and the likelihood of a patient presenting with CCP after accounting for the effects of other variables in the model shown (Table 9). Here are the findings from the multivariate logistic regression analysis:

Age

Each additional year of age was associated with a 5% increase in the odds of having CCP (Odds Ratio [OR] = 1.05, p < 0.001). This suggests that older patients are more likely to have chest pain of cardiac origin.

Smoking

Patients with a history of smoking were found to be more than twice as likely to present with CCP compared to non-smokers (OR = 2.22, p < 0.001). This aligns with the well-known cardiovascular risks associated with smoking.

Diabetes

Having diabetes increased the likelihood of CCP by 57% (OR = 1.57, p = 0.003). Diabetes is a significant risk factor for coronary artery disease, which could explain this association.

Hypertension

Hypertension was associated with an 82% increase in the odds of having CCP (OR = 1.82, p < 0.001), further underscoring its role as a contributory factor to cardiac conditions.

Family History of IHD

A family history of IHD increased the chances of CCP by 42% (OR = 1.42, p = 0.01), indicating a genetic or familial predisposition to cardiac-related chest pain

**Table 9:** Multivariate analysis determining individual predictors and their significance

Variable	Coefficient	Odds Ratio	p-value
Age	0.05	1.05	<0.001
Smoking	0.8	2.22	<0.001
Diabetes	0.45	1.57	0.003
Hypertension	0.6	1.82	<0.001
Family History of IHD	0.35	1.42	0.01

Statistical tools

Each of these factors was evaluated within the model that controlled for the presence of other variables, thus providing a more accurate picture of their independent effects. The p-values indicate that these associations were statistically significant, meaning that the likelihood of these findings being due to chance is very low.

The coefficients provided in the regression model are logarithmic transformations of the odds ratios. For example, the coefficient for age (0.05) means that for each one-year increase in age, the log odds of having CCP (as opposed to NCCP) increase by 0.05. The odds ratio is the exponentiated coefficient, interpreted as the multiplicative effect on the odds of the outcome for each unit increase in the predictor.

## Discussion

Our study's multivariate analysis revealed that age, smoking, diabetes, hypertension, and a family history of IHD are significant independent predictors of CCP. Each additional year of age increases the likelihood of presenting with CCP, which is consistent with previous studies indicating that the prevalence of coronary artery disease increases with age [8]. The finding that smokers are over twice as likely to present with CCP compared to non-smokers (OR=2.22, p<0.001) aligns with extensive literature that establishes smoking as a modifiable risk factor for cardiovascular disease [9].

The relationship between diabetes and an increased likelihood of CCP (OR=1.57, p=0.003) in our study echoes the findings of a large cohort that identified diabetes as a strong predictor of coronary heart disease [10]. Similarly, our findings regarding hypertension (OR=1.82, p<0.001) corroborate with other studies that have recognized hypertension as a critical risk factor for developing cardiac-related complications [11]. A family history of IHD increased the chances of CCP by 42% (OR=1.42, p=0.01) in our analysis, which aligns with the literature suggesting a genetic predisposition to cardiovascular diseases [12].

The demographic distribution of chest pain in our study revealed a higher incidence of CCP in older and male patients. This finding is consistent with previous research showing that males are more prone to coronary artery disease, potentially due to protective hormonal factors in pre-menopausal women [13]. The urban predominance (70% urban vs. 60% rural, p=0.034) in CCP patients could be attributed to lifestyle factors associated with urban living, such as diet, stress, and sedentary behavior [14].

Our study's chief complaints and pain characteristics, such as central chest pain, epigastric pain, and left-sided chest pain, provided valuable diagnostic information. Central chest pain was more associated with CCP (60% vs. 40%, p<0.001), which is a well-documented symptom of myocardial infarction [15]. However, the significant report of epigastric pain in the NCCP group (25% vs. 10%, p=0.005) suggests the potential of gastrointestinal etiologies, a common finding in the differential diagnosis of chest pain [16].

The ECG findings from our study were crucial in differentiating CCP from NCCP. ST-segment depression, elevation, and T-wave inversion were significantly associated with CCP, which is a reflection of the diagnostic value of ECG in acute coronary syndromes [17]. A normal ECG was more common in the NCCP group (60%), indicating that a normal ECG does not rule out significant pathology but may point towards non-cardiac causes [18].

The outcome analysis indicated that while symptom improvement was high across both groups, the NCCP group had a slightly higher rate of improvement (87% vs. 78%, p=0.013). This could be due to the less severe nature of non-cardiac conditions or a quicker response to treatment for conditions such as musculoskeletal pain or gastroesophageal reflux [19]. Hospitalization requirements were significantly higher in the CCP group, which is expected given the potential for serious cardiac events requiring close monitoring and intervention [20].

In conclusion, our study provides a nuanced understanding of the clinical presentation of chest pain in a tertiary care setting in North India. By identifying specific demographic, clinical, and ECG characteristics, along with significant risk factors for CCP, we contribute to the body of evidence that supports the targeted assessment and management of patients presenting with acute chest pain in the emergency department.

Limitations

The study, while providing valuable insights into the incidence and characteristics of atypical chest pain in a North Indian emergency department, is not without limitations. It was a single-center study conducted in a tertiary care hospital in North India, and therefore, the results might have limited generalizability. The specific healthcare practices, patient demographics, and regional health trends of this particular setting may not accurately represent other hospitals, regions, or countries. This limitation is critical when considering the application of these findings to different populations or healthcare systems.

Furthermore, there's a notable selection bias inherent in the study design. The patient sample was confined to individuals who actively sought care in the emergency department. This approach potentially excludes a subset of patients with milder symptoms that might be managed in outpatient settings or those who, for various reasons, did not seek medical attention at all. Such exclusion could lead to an overrepresentation of more severe or acute cases, skewing the understanding of the true spectrum of atypical chest pain presentations.

## Conclusions

The study demonstrates a strong link between traditional cardiovascular risk factors and CCP likelihood in an ED. Key risk factors like age, smoking, diabetes, and hypertension showed a significant association with CCP (p<0.001 for all), aligning with existing literature. Smoking, in particular, emerged as a notable risk, doubling the odds of CCP (OR=2.22, p<0.001), highlighting the need for public health focus on smoking cessation. Demographically, older, urban males were more prone to CCP, suggesting the need for targeted screening in such populations.

Clinically, the patterns of chest pain, especially central chest pain, and dyspnea were indicative of CCP, consistent with acute coronary syndromes. The high incidence of normal ECGs in the NCCP group (60%, p<0.001) underscores the importance of ECG as a diagnostic tool. This study advocates for integrating clinical, demographic, and ECG data in managing acute chest pain, potentially enhancing patient outcomes through timely and precise interventions.
